# Strengthening the Connection Between the Supplemental Nutrition Assistance Program and Farmer’s Markets

**DOI:** 10.5888/pcd13.160186

**Published:** 2016-12-08

**Authors:** Bridget Igoe, Dennis McDermot, Mandy Stahre

**Affiliations:** 1 Office of Healthy Communities, Washington State Department of Health, Olympia, Washington.

**Figure:**
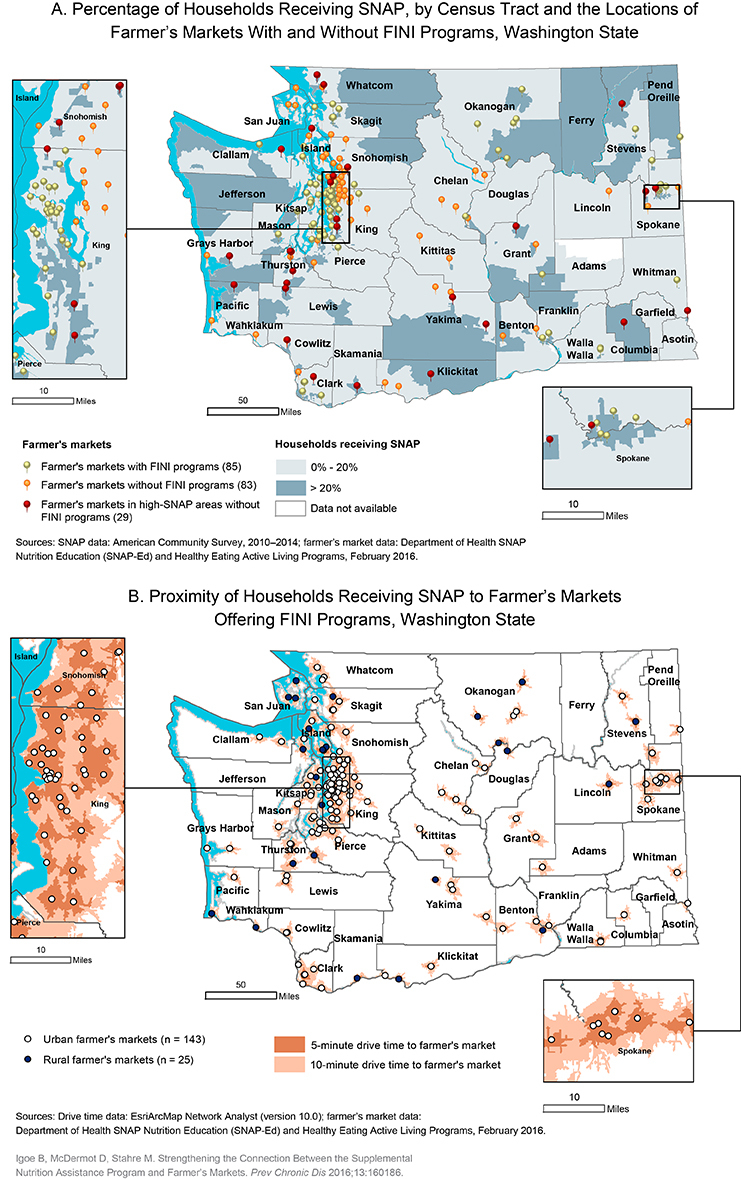
Food Insecurity Nutrition Incentive (FINI) programs incentivize Supplemental Nutrition Assistance Program (SNAP) participants to purchase more fruits and vegetables. The Washington State Department of Health developed these maps of the state to 1) assess the geographic distribution of farmer’s markets with FINI programs in relation to areas with high SNAP populations (>20% of households participate in SNAP) (panel A); 2) estimate the number of SNAP households with reasonable proximity to farmer’s market offering FINI programs (panel B); and 3) identify farmer’s markets that should be prioritized for future SNAP incentive programming.

## Background

At the population level, increased consumption of fruits and vegetables can help to reduce and prevent chronic diseases, including obesity, diabetes, heart disease, and stroke. Addressing affordability of healthier foods, including fresh fruits and vegetables, is especially crucial for socioeconomically disadvantaged populations, who tend to have less healthy diets and higher rates of chronic disease risk than more socioeconomically advantaged groups (1,2). With support from the Centers for Disease Control and Prevention (CDC) and the US Department of Agriculture Food Insecurity Nutrition Incentive (FINI) grant (3), the Washington State Department of Health made it a priority to expand healthy food access and affordability for low-income individuals and families. Washington’s FINI grant for 2015 through 2019 supports cash-value fruit and vegetable incentives offered at the point of sale to participants in the Supplemental Nutrition Assistance Program (SNAP) who shop at participating farmer’s markets and supermarkets. For example, through the FINI program, customers who use their SNAP benefits at participating farmer’s markets receive extra market tokens that can be used like cash to buy more fresh fruits and vegetables at the market. The aim of this GIS Snapshot was to 1) assess the geographic distribution of farmer’s markets with FINI programs in relation to areas with high SNAP populations; 2) estimate the number of SNAP households with reasonable proximity to farmer’s markets offering FINI programs; and 3) identify farmer’s markets where future SNAP incentive program efforts should focus to reach more SNAP households. This is the first article that uses geographic information systems (GIS) data to evaluate proximity to farmer’s markets that offer nutrition incentives to SNAP participants.

## Methods

Data on the prevalence and number of SNAP households by census tract were obtained from the US Census Bureau American Community Survey (ACS) from 2010 through 2014. A high-SNAP area was defined as a census tract in which more than 20% of households participate in SNAP. To identify farmer’s market locations, 2014 program data from the Department of Health’s SNAP Nutrition Education (SNAP-Ed) program were used and updated to capture data on markets participating in the FINI grant as of February 2016. Farmer’s markets with and without FINI programs were identified by whether their location was in a high-SNAP area.

Reasonable proximity to farmer’s markets was defined as residing within a 5-minute or 10-minute drive of a market and was assessed for urban and rural areas. Esri ArcMap (version 10.0) Network Analyst was used to generate 5-minute and 10-minute service areas around farmer’s market locations. Service areas consisted of the area extending 100 m from the centerline of all street segments less than the specified drive time from a farmer’s market. For each service area, urban and rural census blocks were identified that intersected the service area (inside) and those that did not intersect the service area (outside). Urban census blocks were urbanized areas or urban clusters as designated by the US Census Bureau for 2010. Rural census blocks were those that were not urban. To estimate SNAP households by census block, total population was multiplied at the block level by the census tract level fraction of SNAP households based on 2010 through 2014 data from the ACS. We then identified SNAP households for urban and rural census blocks inside each service area.

## Main Findings

Panel A shows that farmer’s markets with FINI programs were broadly distributed across Washington State in high-SNAP areas. Of 168 farmer’s markets, 85 markets (50.6%) are projected to offer fruit and vegetable incentives to SNAP participants by 2019 through the Department of Health’s FINI grant. Of these 85 markets, 33 (39%) were in high-SNAP areas and 75 (88%) were in urban areas. There were 83 farmer’s markets (49%) not funded by the Department of Health’s FINI grant. Of these, 29 (35%) were in high-SNAP areas and 68 (82%) were in urban areas.

Panel B together with the data in the Table demonstrate the urban-rural differences in the percentage of SNAP households within 5-minute to 10-minute drive times to farmer’s markets with and without FINI programs. In urban areas, the percentage of SNAP households within 5-minute to 10-minute drive times was larger for farmer’s markets with FINI programs than those without (5 minutes: 58.9% vs 42.6%, respectively; 10 minutes: 65.6% vs 56.0%, respectively). However, in rural areas there was a larger percentage of SNAP households within 5-minute to 10-minute drive times to farmer’s markets without FINI incentives (approximately 60%) compared to those with FINI incentives (approximately 40%).

**Table:** Urban-Rural Differences in Households Participating in the SNAP^a^ Within 5-Minute and 10-Minute Drive Times to Farmer’s Markets With and Without FINI Program^b^ Incentives, Washington State

Location	No. (%) Within 5-Minute Drive Time		No. (%) Within 10-Minute Drive Time	
	With FINI Incentives	Without FINI Incentives	With FINI Incentives	Without FINI Incentives
Urban	125,712 (58.9)	90,882 (42.6)	140,005 (65.6)	119,568 (56.0)
Rural	2,178 (40.0)	3,304 (60.7)	2,194 (40.3)	3,315 (60.9)

Abbreviations: FINI, US Department of Agriculture Food Insecurity Nutrition; SNAP, Supplemental Nutrition Assistance Program.

a SNAP data are from the American Community Survey, 2010–2014.

b Farmer’s market data are from the Washington State Department of Health, 2016.

A visual scan of Panel A indicates many potential farmer’s markets where SNAP nutrition incentive programming could be expanded, especially to markets in high-SNAP areas. Additionally, results from the network analysis (panel B and the Table) will enable program planners to take a closer look at the percentage of SNAP households within the 5-minute and 10-minute drive times to farmer’s markets with and without FINI incentives.

## Action

Geospatial information can be used to assess current and potential reach of programs that aim to increase the affordability of fruits and vegetables for SNAP participants offered in retail locations serving large SNAP populations. We assessed the geographic distribution of farmer’s markets offering such incentives in relation to high-SNAP areas, urban and rural areas, and within specific drive times, indicating opportunities to strategically expand incentive programming to markets that have the highest percentage of SNAP households within their service areas. These maps and the associated network analysis will be used to engage farmer’s markets and local food access program planners in future efforts to expand fruit and vegetable incentives to more markets and in communities with the most SNAP households. This study has several limitations that should be addressed in future analyses. Using geocoded administrative data from the SNAP program, if available, would be preferable to ACS data because SNAP participation is underreported in the ACS (4). Additionally, given the importance of transportation to accessing farmer’s markets (5), household vehicle access, availability of public transportation, and walkability to farmer’s markets should be considered.
