# Erratum, Vol. 13, December 8 Release

**DOI:** 10.5888/pcd14.160186e

**Published:** 2017-03-23

In the article “Strengthening the Connection Between the Supplemental Nutrition Assistance Program and Farmer’s Markets,” the bullets in the key to Panel A in the figure were incorrect. The correction was made to our website on March 10, 2017, and appears online at https://www.cdc.gov/pcd/issues/2016/16_0186.htm. We regret any confusion or inconvenience this error may have caused.

